# Microfluidics combined with fluorescence *in situ* hybridization (FISH) for *Candida spp.* detection

**DOI:** 10.3389/fbioe.2022.987669

**Published:** 2022-09-23

**Authors:** Violina Baranauskaite Barbosa, Célia F. Rodrigues, Laura Cerqueira, João M. Miranda, Nuno F. Azevedo

**Affiliations:** ^1^ LEPABE–Laboratory for Process Engineering, Environment, Biotechnology and Energy, Department of Chemical Engineering, Faculty of Engineering of University of Porto, Porto, Portugal; ^2^ ALiCE–Associate Laboratory in Chemical Engineering, Faculty of Engineering, University of Porto, Porto, Portugal; ^3^ CEFT–Transport Phenomena Research Center, Department of Chemical Engineering, Faculty of Engineering of University of Porto, Porto, Portugal

**Keywords:** FISH, microfluidics, UTI, detection, *C. tropicalis*

## Abstract

One of the most prevalent healthcare-associated infection is the urinary tract infection (UTI), caused by opportunistic pathogens such as *Candida albicans* or non-albicans *Candida* species (NACS). Urine culture methods are routinely used for UTI diagnostics due to their specificity, sensitivity and low-cost. However, these methods are also laborious, time- and reagent-consuming. Therefore, diagnostic methods relying on nucleic acids have been suggested as alternatives. Nucleic acid-based methods can provide results within 24 h and can be adapted to point-of-care (POC) detection. Here, we propose to combine fluorescence *in situ* hybridization (FISH) with a microfluidic platform for the detection of *Candida* spp. As a case study we used *C. tropicalis*, which is reported as the second most common NACS urine isolate obtained from patients suspected with UTI. The microfluidic platform proposed in this study relies on hydrodynamic trapping, and uses physical barriers (e.g., microposts) for the separation of target cells from the suspension. Using a specific peptide nucleic acid (PNA) probe, the FISH procedure was applied onto previously trapped *C. tropicalis* cells present inside the microfluidic platform. Fluorescence signal intensity of hybridized cells was captured directly under the epifluorescence microscope. Overall, the PNA probe successfully detected *C. tropicalis* in pure culture and artificial urine (AU) using FISH combined with the microfluidic platform. Our findings reveal that FISH using nucleic acid mimics (PNA) in combination with microfluidics is a reliable method for the detection of microorganisms such as *C. tropicalis*. As such, this work provides the basis for the development of a POC detection platform in the future.

## 1 Introduction

Urinary tract infections (UTI) represent one of the most common healthcare-associated infection across EU countries, accounting for 18.9% of all cases (OECD/European Union, 2018). These infections are caused by microbial pathogens, either bacteria or fungi. While *Candida* species are natural residents of the genitourinary, gastrointestinal tract and skin human flora (Kauffman, 2014), they are also considered as opportunistic pathogens and can cause fungal infections (Richardson, 1991; Fisher, 2011). Infections resulting from *Candida albicans* and non-*Candida albicans Candida* (NCAC) species have increased significantly in the last decade. The presence of *Candida* species in urine–candiduria - is a common clinical finding, particularly in hospitalized patients, mainly with the use of medical devices and/or immunosuppression (e.g., antibiotic therapy, diabetes) (Alvarez-Lerma et al., 2003; Kauffman, 2014; Rodrigues C. F. et al., 2019; Gajdacs et al., 2019). Candiduria denotes a diagnostic and therapeutic challenge for physicians from primary care or infectious diseases, intensive medicine and surgery (Bongomin et al., 2017), because it may be linked to numerous conditions that require careful interpretation, from sample contamination to UTI or even disseminated candidiasis. The identification of *Candida* isolates to species level is necessary due to different antifungal susceptibility patterns, which is important for administration of appropriate therapeutic strategy (Kauffman et al., 2011). *Candida albicans* is the most prevalent species isolated from urine samples (51.8%) (Kauffman et al., 2000; Fisher, 2011). However, recent studies indicate increased incidence of NCAC in clinical samples (Goyal et al., 2016; Taei et al., 2019). For instance, *Candida tropicalis* is reported as the second most common NCAC (14.3%) identified in urine samples obtained from patients suspected with UTI (Gharanfoli et al., 2019). It is also a frequent isolate detected in urine samples of both inpatients (8.95%) and outpatients (8.69%) (Gajdacs et al., 2019).

In clinical microbiology, urine culture methods are used for UTI diagnosis. At first the causative microorganism is identified from urine culture which takes 18–48 h. Afterwards, the antimicrobial susceptibility testing (AST) is performed, which takes additional 24 h (Wilson and Gaido, 2004; Davenport et al., 2017). While these culture methods are used for routine urine examination (Aspevall et al., 2001) due to their cost-effectiveness and specificity, although some limitations are also present (Kauffman, 2014). Besides standard urine culture-based methods, *Candida* spp. Can be detected through microscopy visualization with the aid of Gram staining (e.g., *Candida albicans* appear in budding yeast 4–10 μm in diameter) (Kauffman et al., 2011). Other diagnostic techniques include the imaging studies, such as ultrasonography, ultrasound or computed tomography urograms (Kauffman et al., 2011; Kauffman, 2014).

This diagnostic delay may eventually result in increased severity of the infection. As such, molecular detection methods that rely on proteins or nucleic acids have also been suggested for routine urine analysis. These methods identify microorganisms at the species level within 24 h, which is important for selecting appropriate therapeutics. One example of a molecular method is fluorescence *in situ* hybridization, that relies on fluorescently labelled nucleic acids [DNA, RNA or nucleic acid mimics (NAM’s)] probes (Almeida et al., 2013c; Cerqueira et al., 2013; Mendes et al., 2016; Ferreira et al., 2017; Azevedo et al., 2019) to bind to target sequences of the microorganism of interest by complementary base pairing (Gall and Pardue, 1969). The hybrid complex can then be visualised directly with an epifluorescence microscope (Moter and Gobel, 2000). The miniaturization concept found application in biotechnology, due to certain advantages such as single-cell analysis or high surface area to volume (S/V) ratio, which results in reduced mass and heat transfer times and shorter diffusion distances (Walker et al., 2004). As such, the biochemical reaction time is improved which is important for reducing the overall diagnostic time (Asghar et al., 2019; Sohrabi et al., 2020). Also, upon microfluidics integration with molecular methods, the amount of sample required is reduced and doesn’t compromise the sensitivity and specificity of the system (Hsieh et al., 2022). Moreover, for sample analysis, different visualization devices can be combined with microfluidic platform, allowing spatiotemporal resolution and high detection efficiency (Fan et al., 2021). While other study developed mobile platform with a sensitivity comparable to that of a conventional microscope (Muller et al., 2018). Microchannel geometries are also prone to massive parallelization, allowing high-throughput analysis (Mach and Di Carlo, 2010). Equally important, microfluidic platforms allow to concentrate and separate target microorganisms from biological fluids, thus reducingcircumventing lengthy culture times (Wang et al., 2012; Beech et al., 2018). A number of studies have explored hydrodynamic filtration (Yamada and Seki, 2005), deterministic lateral displacement (DLD) (Inglis et al., 2011), microfiltration (Ji et al., 2008) methods, which allow to separate or enrich microorganisms. These methods do not require any external force field (passive cell separation), making them simple, low-cost and label-free, which simplifies the overall procedure (Zhou et al., 2019). Such passive separation techniques rely on inherent hydrodynamic forces, channel geometries and physical obstacles, such as micropost arrays, microfiltration, microwells and chambers (Luan et al., 2020). The use of these techniques eliminates the need for sophisticated and expensive devices. Hydrodynamic cell trapping has shown to be applicable for single cell imaging and quantification (Park et al., 2011), microorganism enrichment (Whang et al., 2018) and size-selective trapping and sorting (Kim et al., 2014).

The FISH method robustness and implementation as molecular diagnostic tool greatly improves when using NAM’s, which have enhanced sensitivity and specificity, when compared to DNA or RNA probes (Nacher-Vazquez et al., 2022). Moreover, when combined with microfluidics, the analysis time is reduced when compared to standard FISH method (Chien-Hsuan et al., 2013). In spite of its great potential, the integration of FISH and microfluidics has been achieved in a limited number of works (Ferreira et al., 2017; Nguyen et al., 2017; Lee et al., 2022), especially for the diagnostics of fungal infections (Hussain et al., 2020). Therefore, the goal of this work is to develop a microfluidic platform combined with FISH for the detection of *Candida* spp. The proposed method was tested using *C. tropicalis* as a case study.

## 2 Materials and methods

### 2.1 Cell culture maintenance and microbial cell suspension preparation

*C. tropicalis* reference strain (ATCC 750) from the American Type Culture Collection was used in this work. For inoculum preparation, cells were grown overnight (≈16 h) at 37C° and 120 rpm, under aerobic conditions. The growth rate under these conditions was determined by measuring optical density (OD 600 nm) (VWR V-1200 spectrophotometer, United States) over time. Subsequently, cell concentration was adjusted by OD for a final single cell concentration of 1 × 10^8^ cells/ml or 1 × 10^5^ cells/ml (for artificial urine (AU)). To assess cultivability, 1:10 dilutions were prepared in Phosphate Buffered Saline (PBS, 180 mM NaCl, 3 mM KCl, 9 mM Na_2_HPO_4_.2H_2_O, and 1.5 mM KH_2_PO_4_, pH 7.4) and plated onto SD agar plates and incubated overnight, at 37°C, under aerobic conditions (Rodrigues et al., 2018). Then, colony forming units (CFU) were counted to confirm microbial cell concentration (CFU/ml). Finally, 1 ml of *C. tropicalis* cell suspension was centrifuged at 13.000 g for 10 min and resuspended in 1 ml of PBS before proceeding to fluorescence *in situ* hybridization and microfluidic experiments.

### 2.2 Microfluidic platform development

**FIGURE 1:**
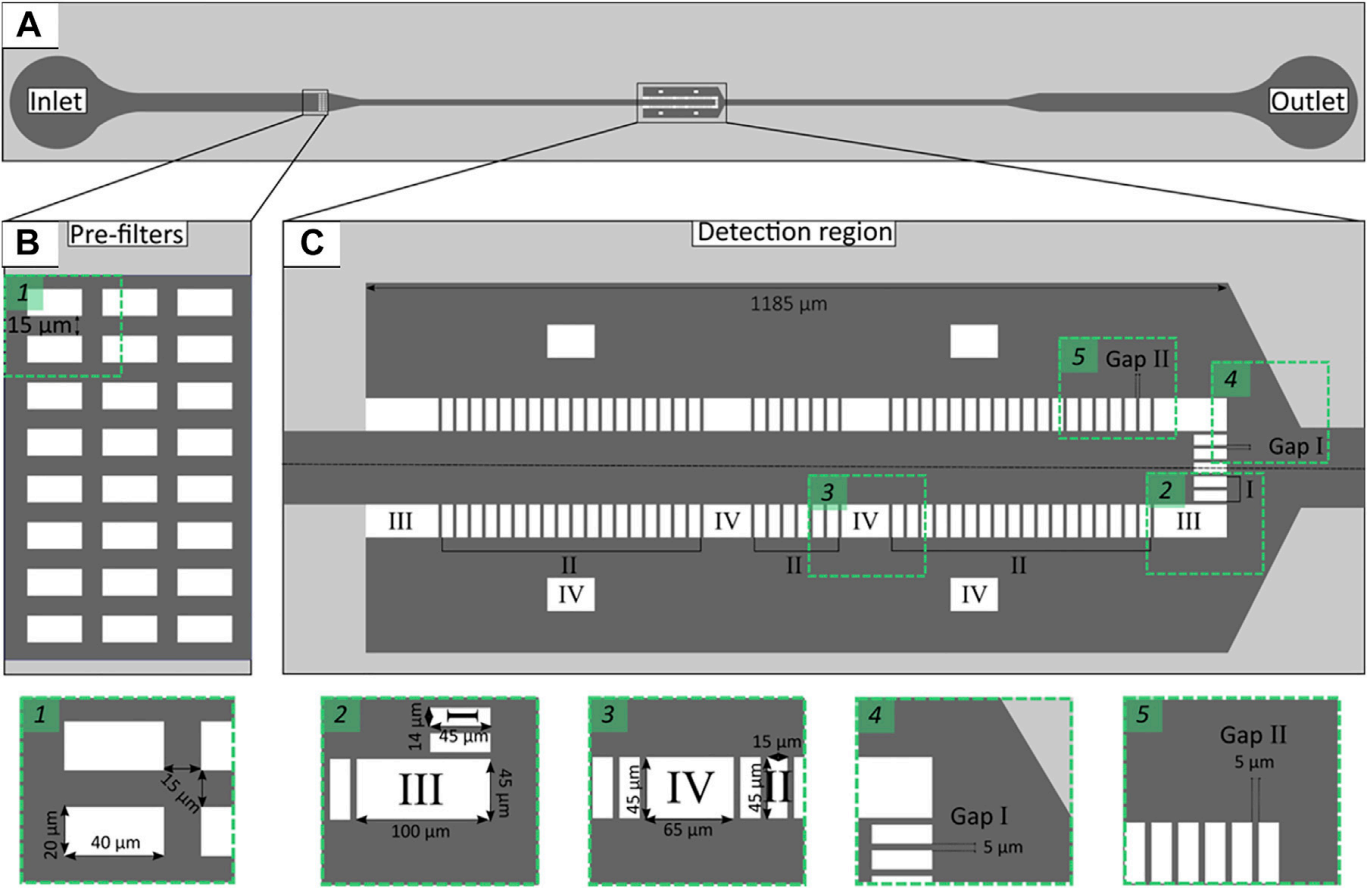
Schematic representation of microfluidic channel layout with single inlet and outlet **(A)**. Enlarged view representing pre-filters **(B)** and the detection region **(C)** containing microposts of different geometries: front (Micropost I), lateral (Micropost II) and support (Micropost III and IV). *Rectangles 1–5* enlarged microchannel layout sections.

The microchannel geometry was designed using AutoCAD 2013^®^ software (Autodesk Inc., United States) and then a silicon master mold was fabricated at the International Iberian Nanotechnology Laboratory-INL facilities (Braga, Portugal) combining direct laser write lithography and deep reactive ion etching. The microchannel layout proposed in this study consisted of an inlet, an outlet and a widened detection region (500 μm) (Figure 1A). The depth of the microchannel was set to 30 μm, because low *height*/*width* ratio provides better micropost stability (Ferreira et al., 2017). Also, the depth of 30 μm was calculated to be the maximum value at which low *height*/*width* ratio is still maintained. This is an important parameter, as on one hand low *height*/*width* ratio provides better micropost stability and on the other high depths maximise the flow rate.

In comparison to a previous design (Ferreira et al., 2017), three rows of pre-filters (Figure 1B) were introduced to separate target microorganisms from particles or other cells that may be present in clinical samples (e.g., blood or urine). The detection region consisted of different micropost geometries designed to trap cells larger than 5 μm (such as *C. tropicalis*) (Figure 1C, Micropost I and II), while larger microposts provided structural support to the larger cross-sectional area of the microchannel (Figure 1C, Micropost III and IV). The gaps were set to 5 μm throughout the whole trapping array (Figure 1C, Gap I and II).

#### 2.2.1 Microchannel fabrication

The microchannels were produced using the soft-lithography method–a replication of the silicon mold. At first the master mold was placed in a laminar flow chamber with a few drops of trichlorosilane (UCT Specialities, United States) for 1 h. The vapour of trichlorosilane allows an easier removal of the elastomer block (Jung et al., 2005). The two-part polydimethyl-siloxane (PDMS) silicone elastomer kit (Sylgard 184; Dow Corning, United States) was used to produce the liquid polymer. To produce a negative PDMS slab, 5:1 ratio (5 parts of base polymer by weight to one part of curing agent by weight) and mixed for 10 min with vortex mixer (VV3, VWR) at 2,500 min^−1^. Afterwards, the mixture was placed in a desiccator connected to a vacuum line for degassing to remove air bubbles. Then, the liquid polymer was poured onto a master mold, subjected again to degassing and eventually cured for 20 min at 80°C in the incubator (FD 23, Binder, Germany). Afterwards, the negative PDMS slab containing the microchannels was cut out and peeled off from the master mold. Finally, the inlet and outlet holes were punched with a precision tip (7018178, 20 GA, Nordson EFD, United States). For PDMS-glass bonding, the imprinted surface of the negative PDMS stamp and clean ultra-thin cover glass (631.0178, VWR International) were subjected to oxygen plasma treatment (ZEPTO, Diener electronic GmbH, Germany) at 20 W and 1.2 mBar for 30 s, with subsequent joining of the two surfaces. The PDMS-glass was left in contact for 5 min to form an irreversible bond. Before the oxygen plasma treatment, the cover glass was cleaned with acetone (20063.365, VWR Chemicals, France) then rinsed with high-purity water (HPW) and dried with compressed air.

#### 2.2.2 Microchannel geometrical characterization

First, each fabrication microchannel geometry was inspected visually for any geometrical errors or structural instabilities (e.g., micropost bending) using a microscope (*see section: 2.6.2*).

Afterwards, the experimental geometry characterization was performed. For this, one PDMS slab of each design was cut to obtain the channel cross section and placed downwards onto a glass slide. Then, images were recorded (*see section: 2.6.2*) and subsequently processed with Fiji^®^ (ImageJ.net) software (Schindelin et al., 2012) in order to measure the height (*H*) and width (*W*) of the microchannel (inlet/outlet and micropost array). The characterization was performed in triplicate and measurements were used to calculate the percent error (%) between nominal and experimental values (Supplementary Table S1).

#### 2.2.3 Computational fluid dynamics (CFD)

**FIGURE 2:**
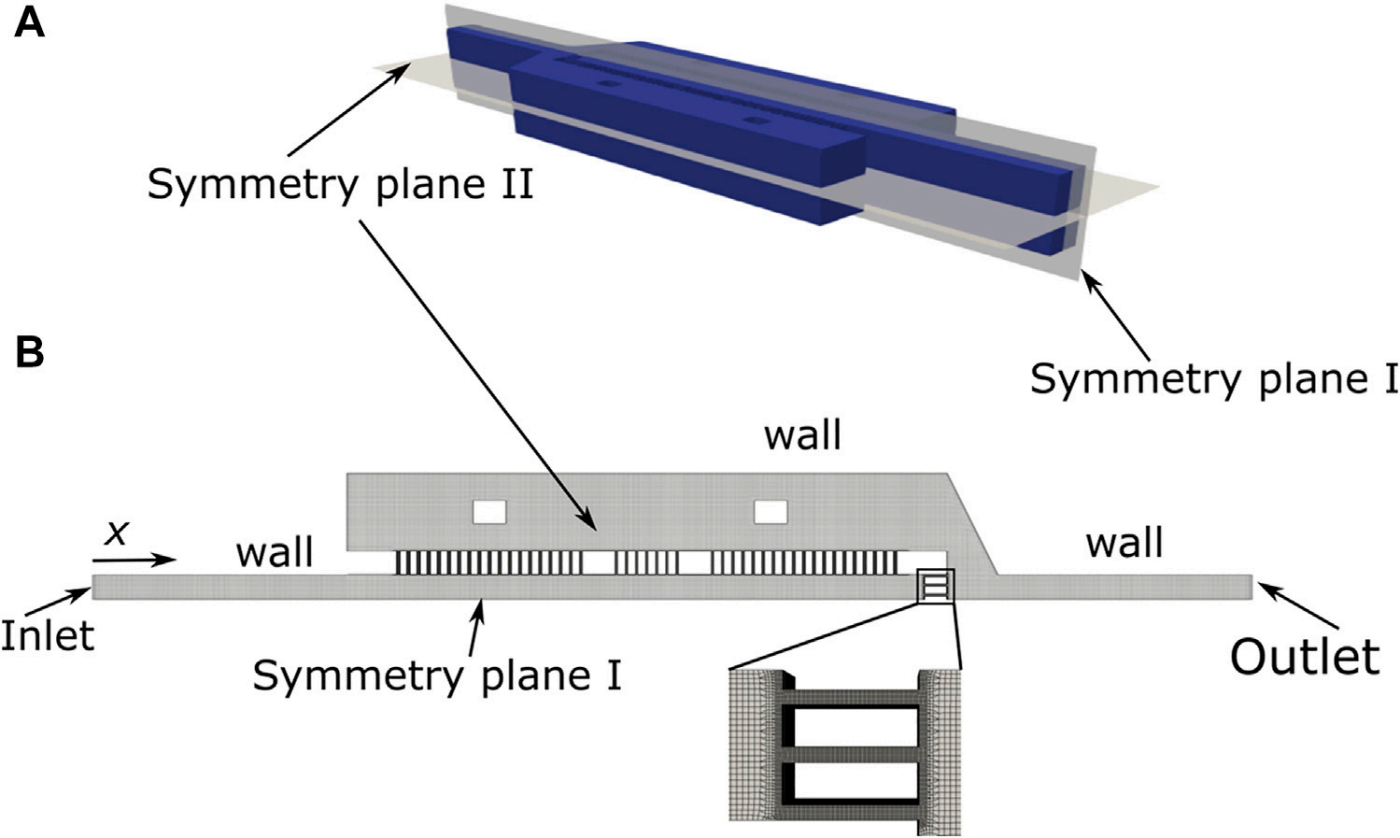
Schematic representation of the device computational domain **(A)** and detailed view of the mesh (inset) **(B)**.

The 2D CAD geometry of the microchannel was extruded to obtain a 3D geometry and was imported to OpenSCAD. In OpenSCAD, the boundaries of the domain were created and exported as STL files. As the geometry is symmetric relatively to two planes (Figure 2A), the flow was solved for 1/4 of the domain (Figure 2B). The boundaries were subsequently named and merged in a single file. This file was used as input to generate the mesh (Figure 2B) using snappyMesh, a mesh generator available on the OpenFOAM framework (version 4.1). The mesh is refined in the gaps (inset in Figure 2B) to ensure an accurate solution of the velocity field.

The flow in the microchannel was solved using the icoFoam solver (OpenFOAM framework, version 4.1), modified to enable an adjustable time step, with the following boundary conditions (Figure 2A): **Inlet:** i) Uniform velocity corresponding to the flow rate of 1 μl/min; ii) pressure gradient normal to the boundary equal to zero; **Outlet:** i) Velocity gradient normal to the boundary equal to zero; ii) pressure set to zero. **Walls:** i) No-slip boundary conditions; ii) pressure gradient normal to the boundary equal to zero. **Symmetry planes:** i) Velocity gradient normal to the boundary equal to zero; ii) pressure gradient normal to the boundary equal to zero. The flow conditions for the simulation are discussed in section 3.1.1. The primary results obtained were the velocity and pressure fields in the domain. These fields were visualized in paraFoam and post-processed to obtain the velocities in the gaps using openFoam post-processing tools.

### 2.3 Fluid handling

**FIGURE 3:**
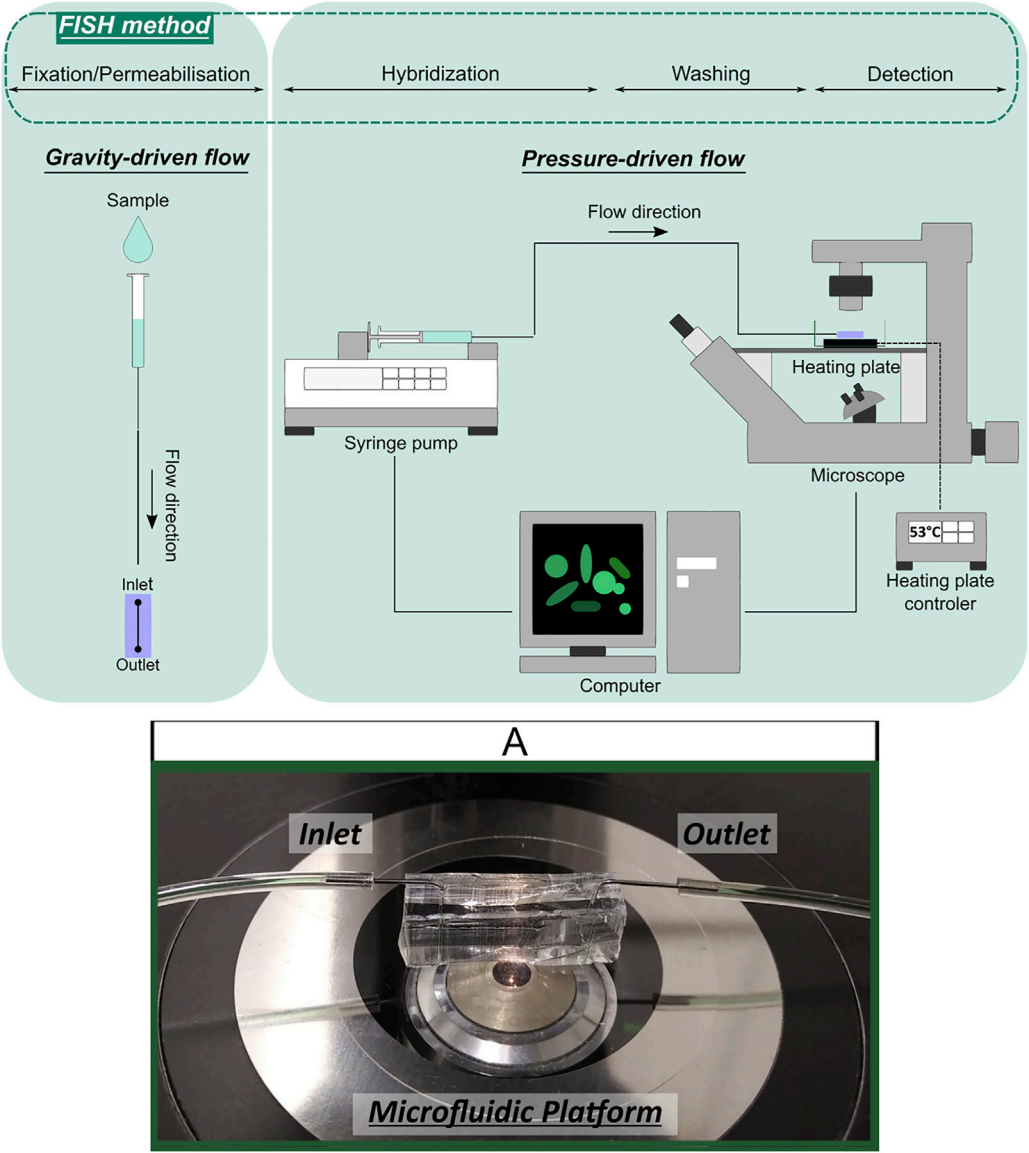
Schematic representation of gravity- and pressure-driven fluid handling systems applied in FISH integration with microfluidics experiments and microfluidic device illustration **(A)**.

For fluid injection in the microchannel and FISH experiments in microfluidic device, gravity- and/or pressuredriven low systems were used (Figure 3). The gravity-driven system was composed of fluid reservoir (e.g., syringe without a plunger) attached to a Tygon microtube (0.44 mm ID) and connected to the inlet of microfluidic platform. The pressure-driven flow system consisted of 500 μl syringe (Hamilton Company, Bonaduz, Switzerland) connected to the inlet of microfluidic platform through Tygon microtube (0.44 mm ID). To achieve pressure-induced laminar flow, the syringe was mounted on a neMESYS low pressure syringe pump (Cetoni, neMESYS syringe pump, Germany), which was controlled through computer software and with the flow rate set to 1 μl/min. In both fluid handling systems, the single microtube was filled with different solutions, which were separated with an air microbubble.

### 2.4 PNA probe design

The PNA-probe targeting *Candida* 18S rRNA was designed by Oliveira (Oliveira, 2018) using BLAST (https://blast.ncbi.nlm.nih.gov/Blast.cgi) and Clustal W (https://www.ebi.ac.uk/Tools/msa/clustalo/) programs for selection and alignment of gene sequences, respectively. Also, the theoretical specificity (%) of 96.04 was calculated. Finally, the PNA-probe containing the following sequence 5′-Alexa488-OO-CACCCACAAAATCAA-3′ was synthesized and HPLC-purified at >90% (BioPortugal, Portugal).

### 2.5 PNA-FISH adaptation to microfluidic environment

#### 2.5.1 PNA-FISH off chip

The hybridization procedure was performed as previously reported (Perry-O’Keefe et al., 2001) with some modifications. A *C. tropicalis* cell suspension (1 × 10^8^ cells/mL) was centrifuged at 10.000 *g* for 5 min (Centrifuge 5,418, Eppendorf, Germany) and resuspended in 500 μl of 4% (w/v) paraformaldehyde (Acros Organics, Belgium) with subsequent incubation for 1 h at room temperature. Then, cells were centrifuged again at 10.000 *g* for 5 min, the pellets re-suspended in 50% (v/v) ethanol (VWR Chemicals, Belgium), and incubated for at least 30 min at -20°C. Subsequently, *C. tropicalis* cells were re-suspended in hybridization solution (pH 7.5) containing 200 nM PNA probe and incubated (FD 23, Binder, Germany) for 1 h at 53°C. The hybridization solution was composed of 10 mM NaCl (VWR Chemicals); 30% (v/v) formamide (VWR, United States); 0.1% (w/v) sodium pyrophosphate (Acros Organics, Spain); 0.2% (w/v) polyvinylpyrrolidone (Sigma-Aldrich, China); 0.2% (w/v) Ficoll^®^ 400 (Sigma-Aldrich, United States); 50 mM di-sodium EDTA (Panreac Quimica, Spain); 50 mM Tris-HCl (Fisher Scientific, United States); 0.1% (v/v) Triton X-100 (Panreac Quimica, Spain) and 10% (w/v) dextran Sulfate (Fisher Scientific, United States) (Cruz-Moreira, 2014). A negative control was performed using hybridization solution without PNA probe. Afterwards, hybridized *C. tropicalis* cells were re-suspended in 500 μl washing solution (pH 10), 5 mM Tris Base (Fisher Scientific, United States), 15 mM NaCl (VWR Chemicals), and 1% (v/v) Triton X-100 (Panreac Quimica), further incubated at 53°C, for 30 min and re-suspended in 500 μl of sterile water. Next, 10 μl of the suspension was injected into microfluidic channel using pressure-driven flow, set at 1 μl/min flow rate (*see section: 2.3*). Cell fluorescent signal was detected by epifluorescence microscopy (*see section: 2.6.1*). In parallel, 20 μl of the previously re-suspended pellets were placed on a glass slide (Hecht Assistent^®^, Germany), dried, mounted with BacLightTM mounting oil (Invitrogene, United States) and covered with a cover slip (24 × 60 mm) for microscopy visualization (Almeida et al., 2013c).

#### 2.5.2 PNA-FISH on chip

The standard PNA-FISH protocol was adapted to work inside the microfluidic device. For this, 20 μL of *C. tropicalis* cell suspension or artificially contaminated urine samples were injected into the microfluidic device by gravity-driven flow (*see section: 2.3*). The artificially contaminated samples were prepared by resuspension of *C. tropicalis* cell pellets (1 × 10^5^ cells/mL) in 1,000 μl of artificial urine (AU) (CaCl_2_, 0.65 g/L; MgCl_2_, 0.65 g/L; NaCl, 4.6 g/L; Na_2_SO_4_, 2.3 g/L; Na_3_C_3_H_5_O(CO_2_)_3_, 0.65 g/L; Na_2_C_2_O_4_, 0.02 g/L; KH_2_PO_4_, 2.8 g/L; KCl, 1.6 g/L; NH_4_Cl, 1.0 g/L; urea, 25.0 g/L; creatinine, 1.1 g/L; and glucose, 0.3% and adjusted to pH 6.5) (Negri et al., 2011). Subsequently, trapped cells were exposed to 20 μl of 4% (w/v) paraformaldehyde and 20 μl of 50% (v/v) ethanol by gravity-driven flow. Next, 10 μl of hybridization solution with PNA probe (200 nM) or hybridization solution alone (control), 10 μl wash buffer and 10 μl of sterile water were introduced through pressure-driven flow system (*see section: 2.3*). The incubation times and temperatures were maintained as described above. For keeping the temperature during the hybridization and washing steps, a heating plate (Leica TPX-TypeF, Leica Microsystems, Germany) was used. Finally, trapped *C. tropicalis* were visualised under the microscope (*see section: 2.6.1*).

### 2.6 Microscopy visualisation

#### 2.6.1 PNA-FISH signal

Images were acquired with a Nikon EclipseT*i* SR inverted epifluorescence microscope (Nikon Instruments, Netherlands) connected to a DS-Ri2 camera (Nikon Instruments). The microscope was equipped with a FITC (fluorescein isothiocyanate) filter sensitive to the Alexa Fluor^®^ 488 fluorophore labelled PNA probe (Excitation 465–495 nm; Barrier 515–555 nm; Dichroic mirror 505 nm). The microscope software NIS-elements 4.13.04 (Nikon Instruments, Amsterdam, Netherlands) was used and parameters such as exposure, gain and saturation were maintained constant in all experiments involving FISH. The acquired images were used for fluorescence signal quantification (*see section: 2.7*).

#### 2.6.2 Geometrical characterisation and cell trapping

Images were recorded with Leica DMI 5000 inverted microscope (Leica Microsystems, Germany) coupled with Leica DFC350 FX camera (Leica Microsystems) and imaging software Leica Application Suite 3.7.0 (Leica Microsystems). To assure the reproducibility and consistency among experimental assays, the microscope parameters (exposure, gain and saturation) were maintained the same in each microscope.

### 2.7 Fluorescence quantification

The fluorescence signal intensity of *C. tropicalis* cells, from 3 independent assays, was quantified from images using Fiji^®^ Software and the procedures described in (Schindelin et al., 2012; Fontenete et al., 2016), with minor modifications. Initially, the original RGB channels (red, green, and blue light) were split and the green channel (where the fluorescence was emitted) was used for fluorescence signal intensity analysis.

The total cell fluorescence, *TCF*, of individual cell was determined as follows:

$$\mathrm{TCF}=\frac{\mathrm{ID}-\mathrm{CA}\times \mathrm{MBF}}{\mathrm{CA}}$$ (1)

where *ID* is the integrated density, *CA* the selected cell area and *MBF* the mean background fluorescence.

This would provide cell size independent quantification and measure of contrast. Then, the mean fluorescence intensity of each cell (considering the cell area), was calculated by:

$$\mathrm{MCF}=\frac{\mathrm{TCF}}{\mathrm{CA}}$$ (2)

where *MCF* is the mean cell fluorescence.

Then mean image fluorescence, *MIF*, was determined as follows:

$$\mathrm{MIF}=\frac{\sum_{i=1}^{N}\mathrm{MC}F_{i}}{\sum_{i=1}^{N}CA_{i}}$$ (3)

where *N* is the number of cells, $MCF_{i}$ is the mean fluorescence of each cell and $CA_{i}$ is the area of each cell.

### 2.8 Statistical analysis

All experiments (“PNA-FISH on chip” *see section: 2.5.2*) were repeated three times in independent assays. Statistical analysis was performed using GraphPad Prism 9 (GraphPad Software, CA, United States). The normality of distribution was tested with D’Agostino-Pearson test, followed by comparison between conditions with the *t*-test. All experimental data are presented as mean ± standard error of the mean (SEM) and statistical significance set at *p* < 0.05.

## 3 Results

### 3.1 Microfluidic channel characterization

**FIGURE 4:**
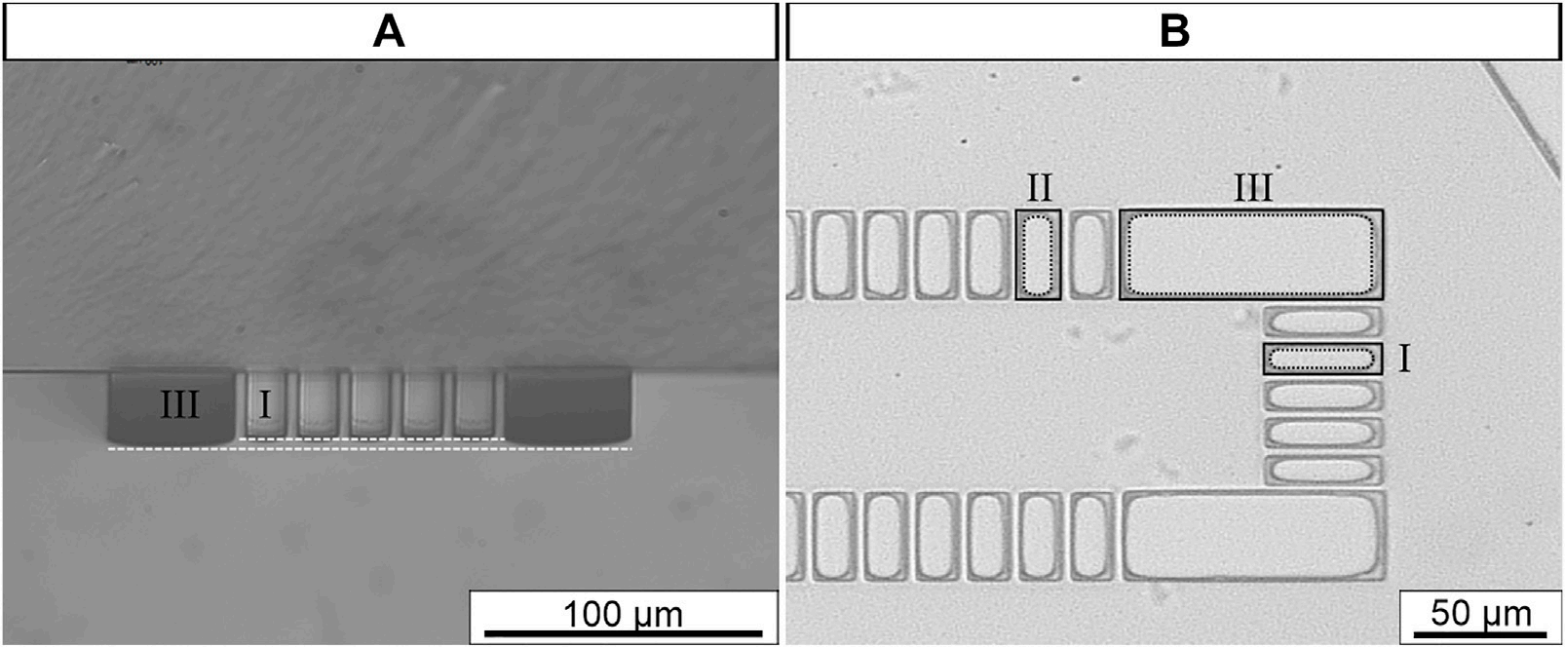
Representative examples of microfluidic channel detection region at cross-section **(A)** and horizontal view **(B)**. *White dashed line* represents micropost height. *Black line* micropost contour; *Black dashed line* micropost contact with glass substrate. Original magnification 400 X **(A)**, 100 X **(B)**.

After fabrication, visual examination of the microfluidic channels revealed that microposts were not visibly bent or deformed and that the microchannel had a well-defined rectangular shape (data not shown) with a vertical side wall (Figure 4). As expected, minor differences in microposts height were observed (Figure 4A, white dashed line). To understand if microposts would fail to bond to glass substrate due to differences in height, we applied oxygen plasma treatment to the microfluidic channel (Ferreira et al., 2017). After treatment it was observed that the surface of all microposts were in contact with the glass substrate (Figure 4B, black dashed line).

Afterwards, the nominal height (*H*) and width (*W*) of the proposed microchannel geometry were compared with the experimental dimensions. Therefore, the microchannel dimensions were measuredat different regions, namely the inlet/outlet and the detection region and the percent error was determined (Supplementary Table S1). It was observed that width percent error ranged between 1.6 and 17.2%. The higher percent error was found to be in the gaps. This was expected because the percent error is generally higher in smaller dimensions (Pinto et al., 2013). Moreover, the height percent error was 10.9–16.0%, suggesting that the master mold height was below 30 μm.

#### 3.1.1 Flow conditions

The next step was to assess hydrodynamic flow conditions by calculating the flow rate (*Q*, µl/min), mean velocity (*V*, m/s) and Reynolds number (*Re*) (Table 1). Since the microchannel has different dimensions of cross-sectional area along the microchannel, the Re number was calculated at different regions. Based on previous studies (Ferreira et al., 2017), an inlet velocity of 0.006 m/s was set, which corresponds to an inlet flow rate of 1 μl/min in a cross-section of 100 μm × 30 μm, where the fluid was assumed to be water (*ρ* = 998.2 kg/m^3^; *μ* = 0.001003 kg m^−1^. s^−1^) (Table 1). As expected, the Re number varied 0.51–0.13 among different regions (inlet/outlet and detection region). These values confirm low *Re*, thus indicating laminar flow and consistency with other microfluidic devices. This data was then used as a starting point to perform computational fluid dynamics (CFD) simulations.

**TABLE 1:** Hydrodynamic flow conditions (nominal).

	Flow rate (*Q*, µl/min)	Velocity (*V*, m/s)	Reynolds number (*Re*)
Inlet/Outlet	1	0.006	0.51
Detection region	1	0.001	0.13

#### 3.1.2 CFD simulations

**FIGURE 5:**
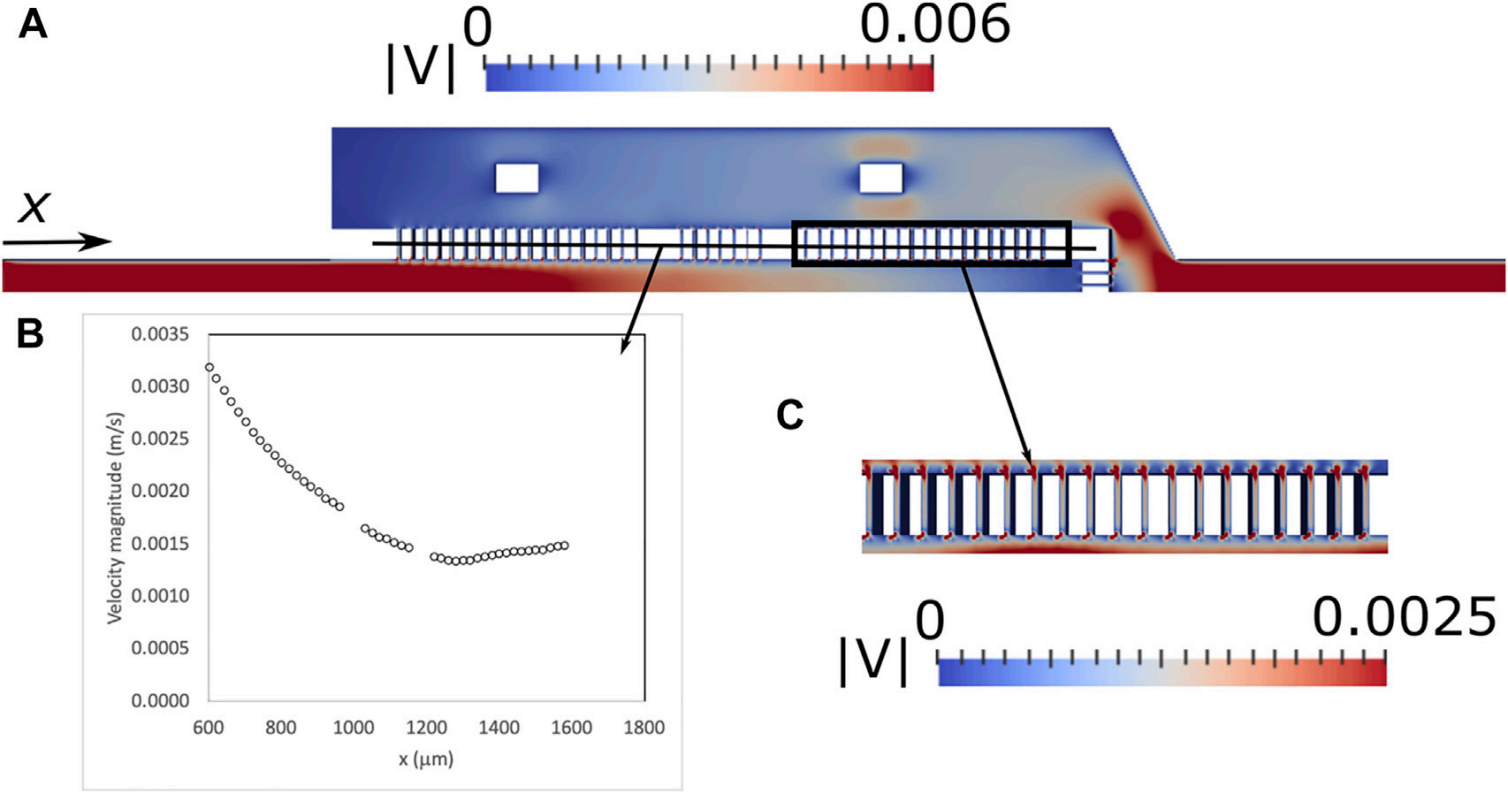
Contours of the velocity magnitude (m/s) for proposed microfluidic devices along the horizontal plane of symmetry (Symmetry plane II, Figure 2A) predicted by the CFD simulations **(A)**, maximum velocity along the lateral gaps **(B)** and detail of the contours of the velocity magnitude in the lateral gaps **(C)**.

The results obtained by the CFD simulations are represented in Figure 5. More specifically, Figure 5A shows the velocity magnitude field at the device horizontal plane of symmetry (Symmetry plane II, Figure 2A). When the fluid enters the trapping region the velocity starts decreasing in the retentate side and increases along the *x* axis in the permeate side, due to the fluid flowing through the gaps. The maximum velocity in the gaps decreases along the *x* direction (Figure 5B) in the first two sets of gaps and stabilizes in the third set of gaps. The maximum velocity ranges from 0.0014 m/s to 0.0031 m/s. As shown in Figure 5C, the velocity magnitudes in the lateral gaps are very similar and it can be assumed that the flow does not have preferential paths. The velocity profile of the front microposts is also similar to the velocity profile of the lateral microposts. The maximum velocity ranges from 0.0009 m/s, in the central gaps, to 0.0018 m/s in the lateral gaps.

### 3.2 Integration of FISH method to microfluidic environment

**FIGURE 6:**
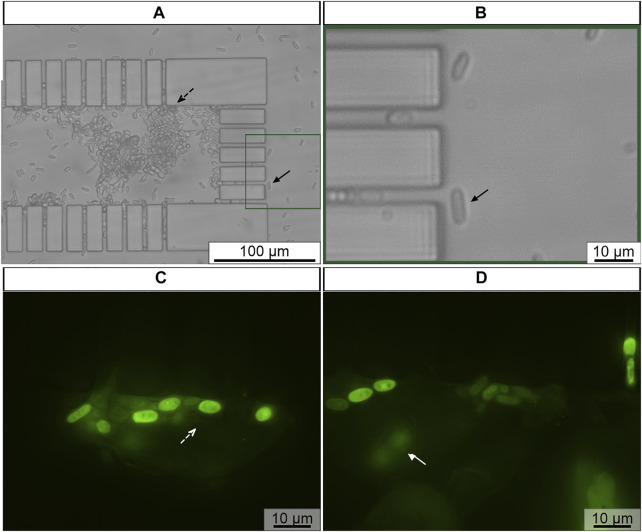
Representative example of *C. tropicalis* subjected to FISH in microchannel **(A,B)** Previously trapped *C. tropicalis* (≈1 × 10^8^ cells/mL) after fixation/permeabilization, **(C,D)** hybridization and washing steps of FISH protocol. The microscope parameters maintained the same. *Black dashed arrow* represents cells at lateral microposts; *Rectangle* enlarged microchannel section; *Black arrow* trapped cell cells at front microposts; *White dashed arrow* fluorescence cells*; White arrow* fluorescence cells in different focus plane. Original magnification 400 X **(A,B)**, 1,000 X **(C,D)**.

In here, the sample was introduced to microfluidic channel using gravity-driven flow, while the fixation, hybridization and washing solutions were injected using the pressure-driven flow system. The experimental set-up used in these assays is outlined in Figure 3. At first, using gravity-driven flow, viable *C. tropicalis* cells were introduced into the microfluidic channel, so that they could be retained along the microposts. Then, using the same flow system, the fixation/permeabilization solutions of the FISH protocol were applied onto trapped *C. tropicalis* cells. During this step the microorganism was permeabilized while preserving the morphological structure of the cells, so that the probe can be internalized in the hybridization step. Cell trapping was confirmed with bright field microscopy (Figures 6A,B). *C. tropicalis* was mainly trapped along the lateral (Figure 6A, black dashed arrow) and front microposts (Figures 6A,B, black arrow). Subsequently, using pressure-driven flow, subsequent hybridization and washing steps were performed. Overall, *C. tropicalis* cells revealed strong fluorescence signal. This confirmed that the PNA probe successfully hybridized to the target microorganism and that FISH steps were performed correctly in the microfluidic environment. Some *C. tropicalis* cells were observed in different focus planes (Figure 6D, white arrow) which may partly explain differences in the fluorescence signal of different cells. Because the microchannel height (≈30 μm) is higher that *C. tropicalis* cell dimension, single or multiple cells can be found in different positions along the microchannel height. As such, images should be acquired at different focus planes.

**FIGURE 7:**
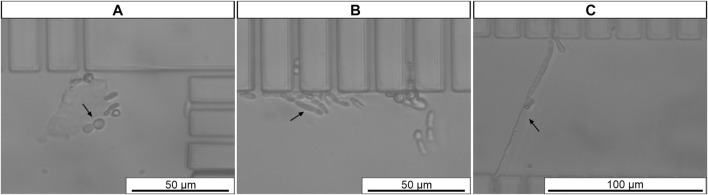
Morphological growth forms of *C. tropicalis*: **(A)** yeast, **(B)** pseudohyphae, **(C)** hyphae. The microscope parameters maintained the same. *Black arrow* represents trapped cell. Original magnification 400 X **(A–C)**.

Different morphological growth forms were observed among *C. tropicalis* cells during integration studies [i.e., yeast, hyphae, pseudohyphae (Figures 7A–C)]. Islam *et al.* (Islam et al., 2020) also assessed *Candida* spp. Cell morphology before microfluidic experiments. In the case of *C. tropicalis* they revealed several morphologies: first spherical (with larger diameter of *x* = *y* = 5.98 ± 0.75 μm) and then pseudosphere with ellipsoidal morphology (length of the pseudohyphae ranged from 7 to 27 μm with an average width of 1.89 ± 0.4 μm). These findings are in the agreement with our observations. Thus, it is possible that elongated filamentous *C. tropicalis* cells may pass through the gaps because of their orientation (e.g., minor length) when flowing in the microchannel.

### 3.3 Microfluidic platform validation in artificially contaminated samples

**FIGURE 8:**
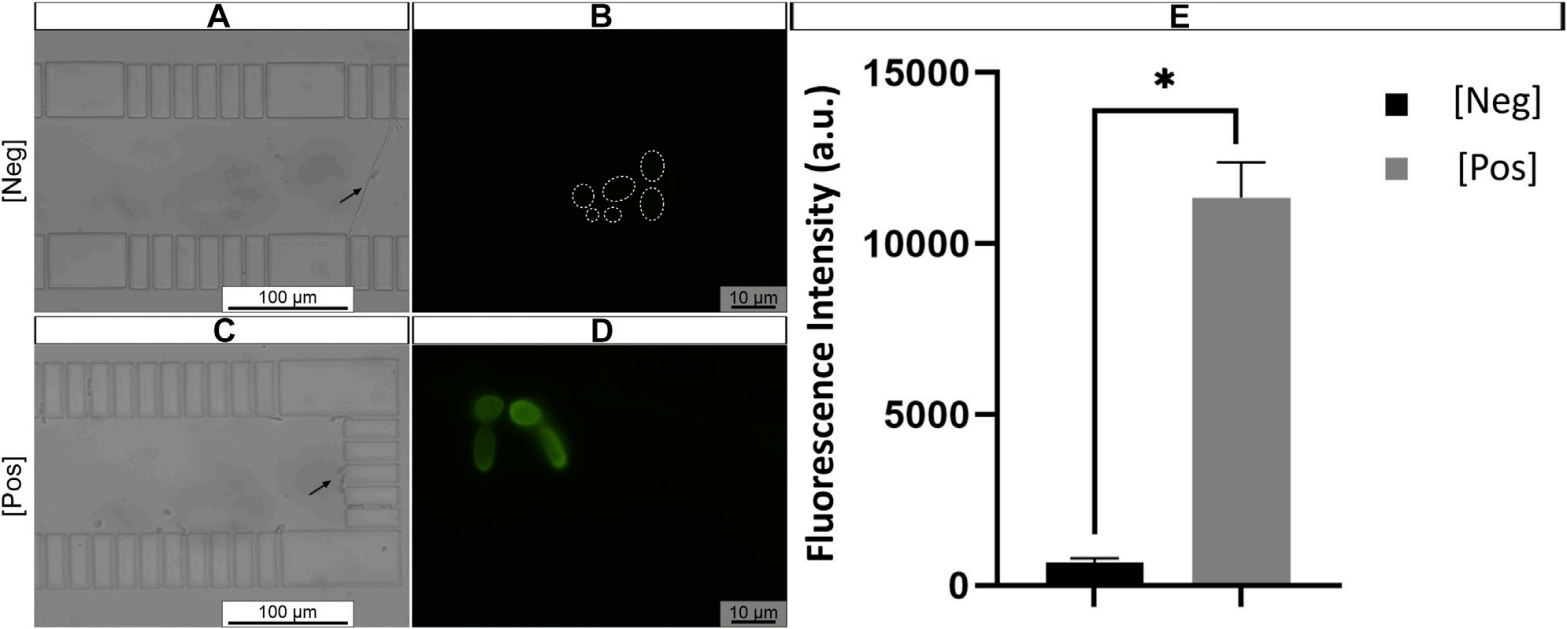
Representative example of artificial urine (AU) contaminated with *C. tropicalis* subjected to microchannel integrated FISH method **(A,C)**. Previously trapped *C. tropicalis* (≈1 × 10^5^ cells/mL) after fixation/permeabilization step. And subjected to hybridization solution (HS) alone (control, *dashed circles*) **(B)** or **(D)** PNA probe suspended in HS (200 nM). The microscope parameters were kept the same. *Black arrow* represents trapped cell; *Dashed circle* hybridized cell contour. Original magnification 400 X **(A,C)**, 1,000 X **(B,D) (E)** The fluorescence signal quantification of *C. tropicalis*. The data is shown as mean fluorescence intensity (arbitrary units a. u.) ± SEM. *p*>0.05: * vs HS alone [Neg], HS + PNA probe [Pos].

Finally, the proposed method was tested in artificially contaminated samples, where AU was contaminated with *C. tropicalis* at 10^5^ cells/mL, a representative concentration for UTI (Fisher et al., 2011). At first, 20 μl of AU contaminated with *C. tropicalis* was introduced to the microfluidic channel. Subsequently, the solutions of FISH protocol were applied sequentially onto already trapped *C. tropicalis* cells using the same fluid handling system as described previously. A negative control with *C. tropicalis* exposed to HS alone was included. Finally, cell trapping was confirmed with bright field microscopy (Figures 8A,C, black arrow). Overall, strong fluorescence signal of *C. tropicalis* cells was observed (Figure 8D) when subjected to PNA probe, compared to cells hybridized in HS alone (Figure 8B, dashed circle). Additionally, the fluorescence signal quantification results revealed that the signal intensity was significantly higher in *C. tropicalis* cells hybridized with probe, when compared to cells subjected to hybridization solution alone (Figure 8E). These findings corroborate with previous observations of visual detection using microfluidic PNA-FISH method (Figure 8D).

## 4 Discussion

The proposed microfluidic channel was fabricated using the silicon-based organic polymer–PDMS. This material is widely used in biological studies (Ferreira et al., 2017) since it presents several advantages, such as biocompatibility, optical transparency, low-cost, and rapid prototyping (Torino et al., 2018). PDMS is composed of -O-Si(CH_3_)_2_- repeating units and the CH_3_ is responsible for hydrophobic surface properties. As such, it is difficult to wet with aqueous solutions and may result in microchannel blocking by air bubbles (Sia and Whitesides, 2003). This challenge can be circumvented by exposing PDMS to oxygen plasma treatment, during which the silanol groups (SiOH) are replaced by methyl groups (SiOH_3_) (Duffy et al., 1998; Kim et al., 2004; Bodas and Khan-Malek, 2007). This changes PDMS surface properties to hydrophilic, thus resulting in surface wetting (Bartali et al., 2017). Additionally, PDMS can be joined with different surfaces through reversible or irreversible seals to form closed microfluidic channel (Tan et al., 2010; Xiong et al., 2014). In this study we used the irreversible seal, where the PDMS block and the glass substrate were exposed to plasma oxidation following by immediate bonding forming a closed microchannel. This type of bonding withstands higher pressures (30–50 psi) when compared to the reversible seal (5 psi) (Sia and Whitesides, 2003) and is more suitable for pressure-driven flow systems. Simultaneously, oxygen plasma treatment also was used for PDMS surface hydrophilization.

Flow in the device is crucial to adequate cell sieving. If the flow velocity in the gaps is too high, cells may be dragged through the gaps. One of the goals of CFD simulations was the analysis of possible preferential paths due large asymmetries in flow resistance in the device. Preferential paths lead to high velocities in specific regions of the device, favouring cell dragging through the gaps. CFD results show that the velocity in the gaps is higher in the first set of gaps. To balance the flow between the gaps, the flow resistance must be increased in the gaps with higher velocity by increasing the gap length. Nonetheless, the maximum velocity in the gaps is 0.0031 m/s and in a previous work we showed that the velocity in the gaps can be as high as 0.02 m/s without significant cell dragging (Ferreira et al., 2017).

The dimensional deviations of the fabricated microchannels were also evaluated, as microchannel and gap dimensions impact fluid velocity and sieving performance during experimental assays. For example, non-vertical side walls may result in different velocity contours and streamlines, when compared with numerical simulations (e.g., CFD), which may impact cell trajectories when flowing in microfluidic channel (Karthikeyan et al., 2018). Additionally, the percent error was determined as a measure of fabrication accuracy. Fabrication accuracy was assessed by Sampaio *et al.* (Sampaio et al., 2015) and Lepowsky *et al.* (Lepowsky et al., 2018) in microfluidic chips fabricated by soft-lithography and 3D-printing methods, respectively. For instance, Lepowsky *et al.* reported varying width percent errors from 5.38 to 10.75% in different microchannel regions. These findings are similar to the microchannel proposed in the present study. Overall, the developed microfluidic geometry had minor differences in microchannel height among different microposts which did not prevent from successful microchannel bonding to glass substrate, after oxygen plasma treatment. Therefore, it was assumed that minor differences in micropost heights should not impact overall microfluidic platform performance in further experiments.

The main objective of this study was to integrate microfluidic channel based on hydrodynamic cell trapping with PNA-FISH. This can provide several advantages for UTI diagnostics from clinical urine samples, such as direct target visualisation (Moter and Gobel, 2000) in complex biological matrices (Almeida et al., 2013a; Almeida et al., 2013b), using epifluorescence microscope - a standard equipment found in clinical laboratories. Also, target microorganisms can be detected within a few hours, while the standard culture-based method would require several days (Almeida et al., 2013b). Furthermore, studies showed that the pre-enrichment time can be reduced using PNA-FISH technique (Almeida et al., 2013c; Cerqueira et al., 2020). Nevertheless, the PNA probes can be designed to target microorganisms at species or genus level (Almeida et al., 2013b; Cerqueira et al., 2013; Rocha et al., 2019) and in this case, it is important for the quick identification of *Candida* spp. Isolates and initiation of appropriate treatment (Oliveira Santos et al., 2018; Rodrigues M. E. et al., 2019). Moreover, biological fluids and matrices may contain inhibitory substances, that may interfere with molecular method performance (Morshed et al., 2007; Schrader et al., 2012).

In FISH, one the main challenge is the autofluorescence arising from biological matrices and inorganic debris. This natural fluorescence could impact microscopic sample examination (Moter and Gobel, 2000; Rohde et al., 2015). Clinical samples, such as urine, may exhibit different fluorescence depending on individuals’ health status (Anwer et al., 2009; Spakova et al., 2020). Finally, the microfluidic device can be operated with small volumes (in μl range), resulting in lower costs of probes used in FISH assays.

Despite the successful PNA-FISH procedure integration with a microfluidic platform was attained, some adjustments to microfluidic design should be further considered. For instance, it was observed that some *C. tropicalis* cells pass through the gaps (Figure 6B, black arrow). Kim *et al.* showed that elongated cells moving with the flow in microchannels follow different trajectories than spherical shape cells. Using computational simulations, it was observed, that elongated cell orientation varied through the flipping motion (Kim et al., 2011). Therefore, the microchannel gap size should be adjusted, taking into consideration the width of *C. tropicalis*.

To further decrease the time of the proposed method several strategies could be applied. Such as, optimising laboratory infrastructures and experimental set-up would streamline the process. Also, the pressure-driven flow could be applied for the entire PNA-FISH method in microchannel. Thus, using one type of liquid handling system would simplify the overall procedure. Finally, the incubation times of hybridization and washing steps could be reduced without compromising the fluorescence signal intensity.

## 5 Concluding remarks

The combination of FISH with microfluidics demonstrated number of advantages, such as cell separation from fluid with subsequent pre-enrichment, also reduced reagent consumption and analysis time. However, this integration was achieved in a limited number of works, especially for the diagnostics of fungal infections (Asghar et al., 2019; Hussain et al., 2020). As such, the work presented in this manuscript aimed to develop a microfluidic platform combined with FISH for the detection of *C. tropicalis*. Overall, the obtained results confirm that FISH worked well in microfluidic channels and demonstrated successful *C. tropicalis* detection in biologically-relevant samples, such as AU. Using our proposed PNA-FISH integrated microfluidic platform, *C. tropicalis* was visually detected in AU in 6 h, which is faster than current urine culture method that takes 18–48 h (Wilson and Gaido, 2004; Davenport et al., 2017). Ultimately, this work provides the necessary fundaments towards development of future POC detection platform.

## Data Availability

The original contributions presented in the study are included in the article/Supplementary Material, further inquiries can be directed to the corresponding authors.

## Abbreviations

PNA, FISH Peptide nucleic acid fluorescence *in situ* hybridization; TCF, total cell fluorescence; ID, integrated density; CA, Cell area; MBF, Mean background fluorescence; MCF, Mean cell fluorescence; MIF, Mean image fluorescence; N, Number of cells; $MCF_{i}$ , Mean fluorescence of each cell; $CA_{i}$ , Area of each cell.
