# Supplementary material for: Microfluidics combined with fluorescence in situ hybridization (FISH) for Candida spp. detection
Source: Front Bioeng Biotechnol. 2022 Sep 23;10:987669. doi: 10.3389/fbioe.2022.987669 (PMC9539416; doi:10.3389/fbioe.2022.987669)
Supplement: Supplementary file 1 [file DataSheet1.docx]

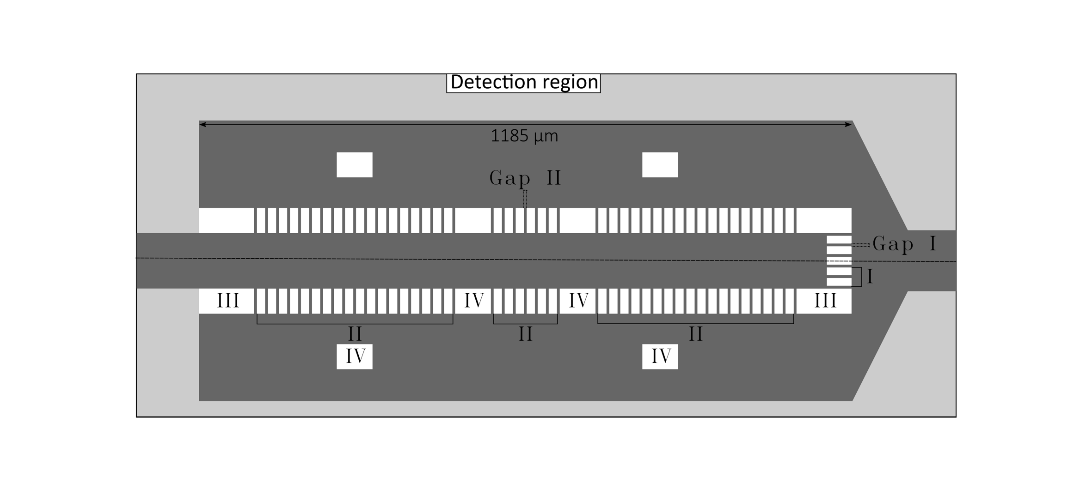
Supplementary Material

**“SUPPLEMENTARY FIGURE S1”** Schematic representation of microfluidic channel detection region containing lateral and front microposts of different geometries (**Micropost I - IV**).


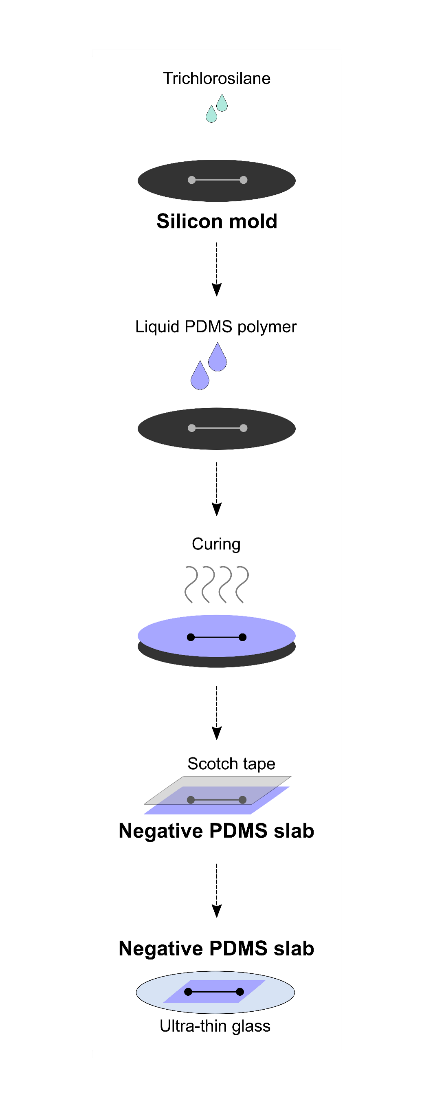
**“SUPPLEMENTARY FIGURE S2”** Schematic representation of microfluidic channel fabrication workflow.


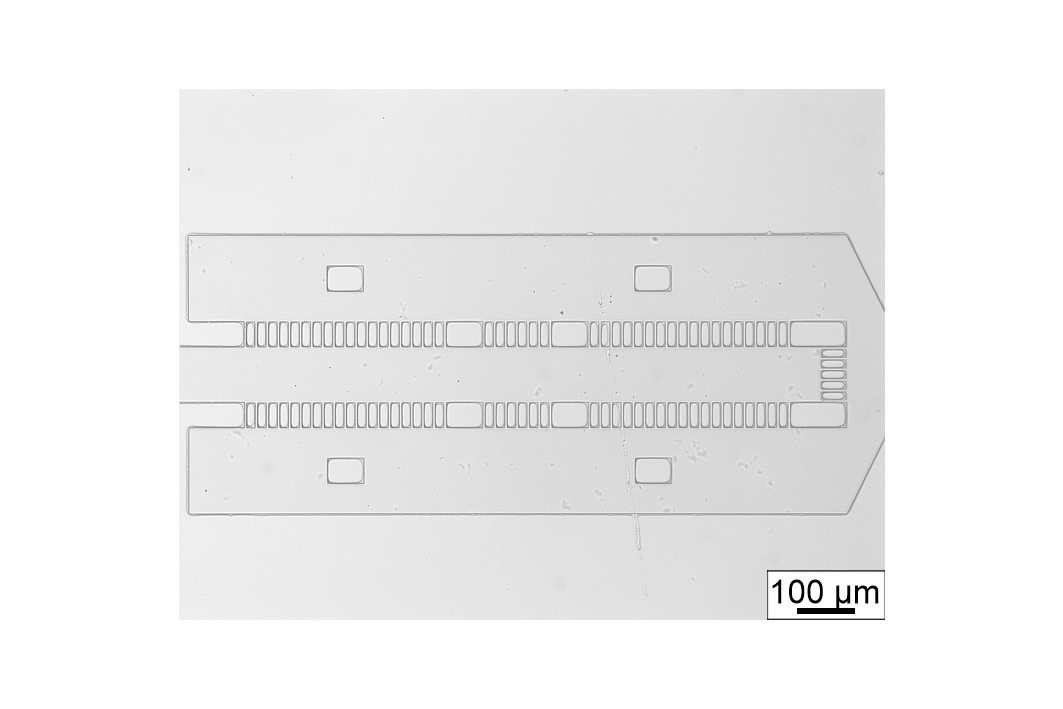
**“SUPPLEMENTARY FIGURE S3”** Representative example of microfluidic channel micropost contact with glass substrate after oxygen plasma treatment. Original magnification, 100X.


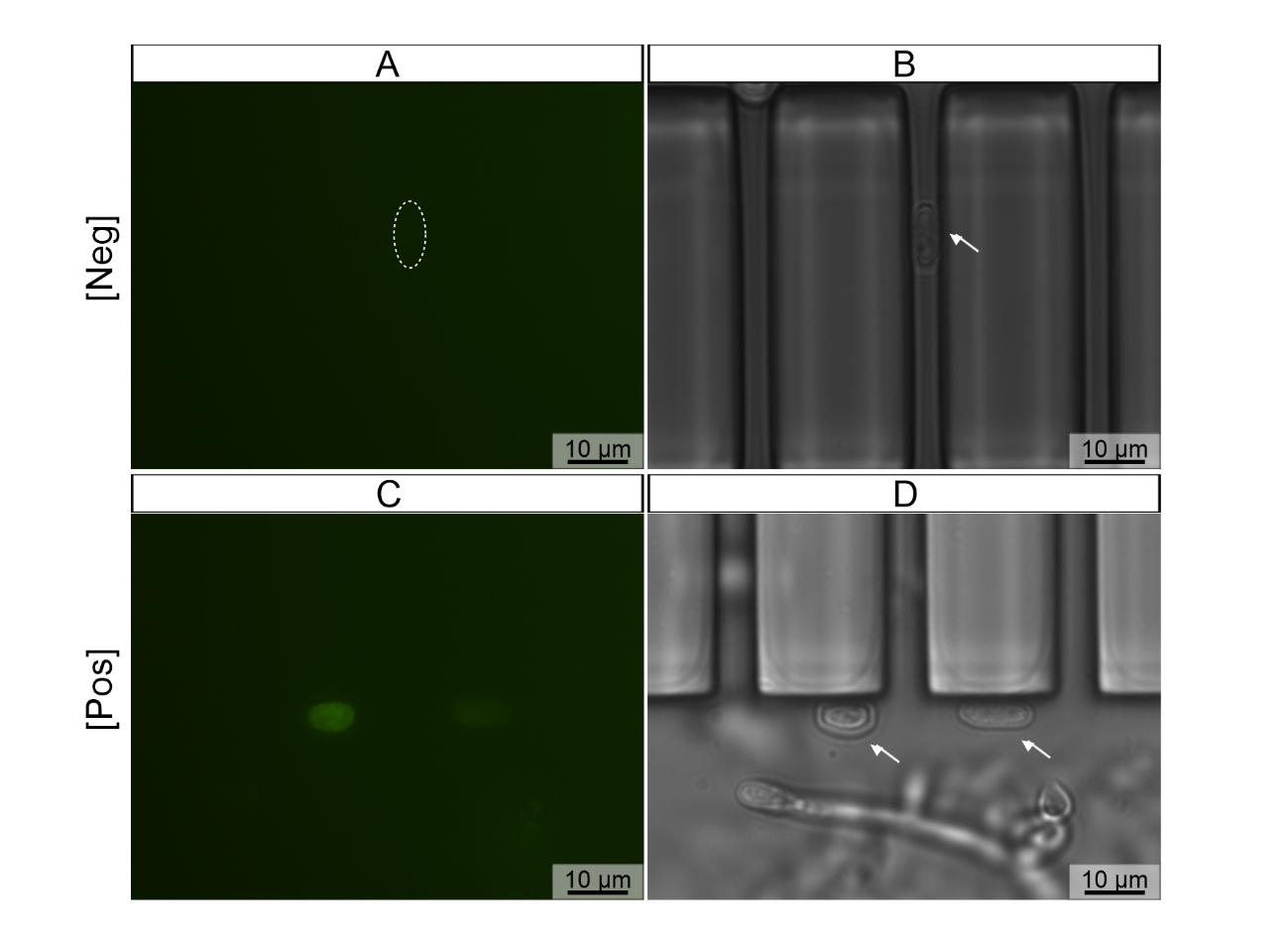


**“SUPPLEMENTARY FIGURE S4”** Representative example of artificial urine (AU) contaminated with *C. tropicalis* subjected to microchannel integrated FISH method. Previously trapped *C. tropicalis* (≈ 1 x 10^5^ cells/mL) after fixation/permeabilization step. And subjected to hybridization solution (HS) alone - [Neg] (control, *dashed circle*) (**B**) or (**D**) PNA probe suspended in HS (200 nM) - [Pos]. The fluorescence (**A** and **C**) and bright-field (**B** and **D**) images were acquired. *White arrow* represents trapped cell; *Dashed circle* hybridized cell contour. Original magnification 1000X (**A** to **D**).

**“SUPPLEMENTARY FIGURE S5”** Representative example of microfluidic channel rectangular shape. *White dashed line* represents rectangular shape of microfluidic channel. *Black dashed* contour
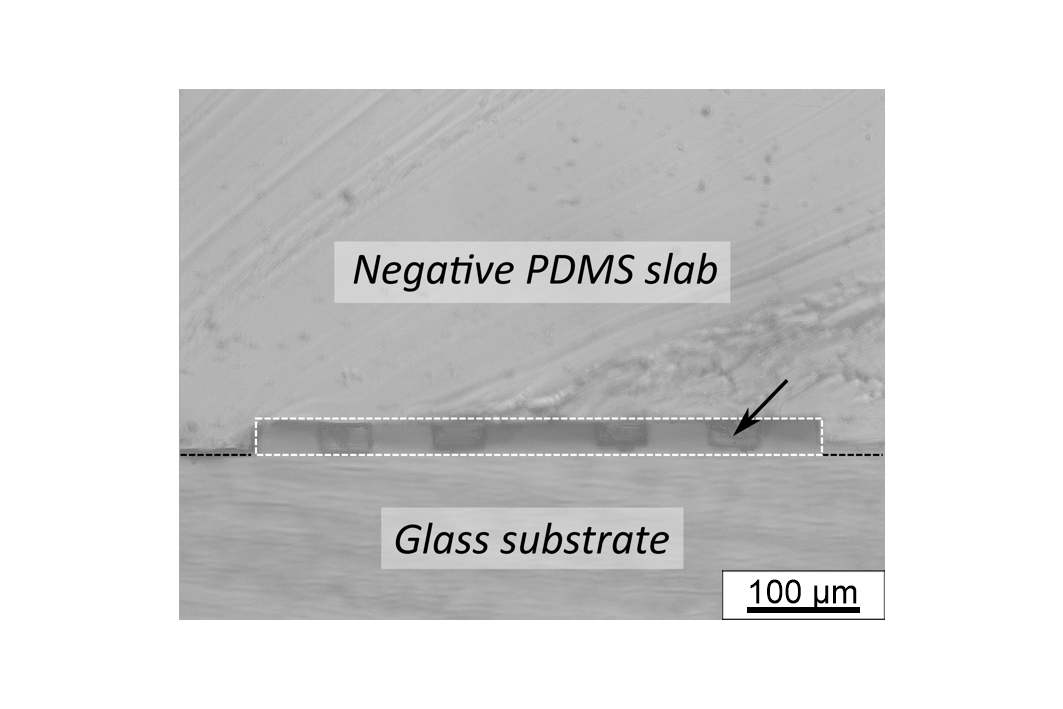
of PDMS contact with glass substrate after oxygen plasma treatment; *Black arrow* micropost; Original magnification, 200X

**“SUPPLEMENTARY TABLE S1”** Nominal and experimental dimensions of microchannel with determined percent error.

|  | **Nominal** | | **Experimental** | | **Percent Error** | |
| --- | --- | --- | --- | --- | --- | --- |
| **Channel** | **Width (µm)** | **Height (µm)** | **Width (µm)** | **Height (µm)** | **Width (%)** | **Height (%)** |
| Micropost I | 14 | 30 | 13.63 | 21.97 | 2.64 | 15.61 |
| Micropost II | 15 | 30 | 14.77 | 21.33 | 1.56 | 16.02 |
| Micropost III | 100 | 30 | 94.97 | 23.31 | 5.03 | 10.92 |
| Micropost IV | 45 | 30 | 43.08 | 22.92 | 4.26 | 15.12 |
| Gap I | 5 | 30 | 4.14 | 22.56 | 17.19 | 15.61 |
| Gap II | 5 | 30 | 4.18 | 22.69 | 16.50 | 16.02 |
| Inlet | 100 | 30 | 88.95 | 22.96 | 11.05 | 11.39 |
| Outlet | 100 | 30 | 88.31 | 21.33 | 11.69 | 12.04 |
